# BMT decreases HFD-induced weight gain associated with decreased preadipocyte number and insulin secretion

**DOI:** 10.1371/journal.pone.0175524

**Published:** 2017-04-26

**Authors:** Saeed Katiraei, Lisa R. Hoving, Lianne van Beek, Sharida Mohamedhoesein, Françoise Carlotti, Janna A. van Diepen, Patrick C. N. Rensen, Mihai G. Netea, Ko Willems van Dijk, Jimmy F. P. Berbée, Vanessa van Harmelen

**Affiliations:** 1 Department of Human Genetics, Leiden University Medical Center, Leiden, The Netherlands; 2 Einthoven Laboratory for Experimental Vascular Medicine, Leiden University Medical Center, Leiden, The Netherlands; 3 Department of Medicine, division of Nephrology Leiden University Medical Center, Leiden, The Netherlands; 4 Department of Internal Medicine, Radboud UMC, Nijmegen, The Netherlands; 5 Department of Medicine, division of Endocrinology Leiden University Medical Center, Leiden, The Netherlands; State University of Rio de Janeiro, BRAZIL

## Abstract

Experimental bone marrow transplantation (BMT) in mice is commonly used to assess the role of immune cell-specific genes in various pathophysiological settings. The application of BMT in obesity research is hampered by the significant reduction in high-fat diet (HFD)-induced obesity. We set out to characterize metabolic tissues that may be affected by the BMT procedure and impair the HFD-induced response. Male C57BL/6 mice underwent syngeneic BMT using lethal irradiation. After a recovery period of 8 weeks they were fed a low-fat diet (LFD) or HFD for 16 weeks. HFD-induced obesity was reduced in mice after BMT as compared to HFD-fed control mice, characterized by both a reduced fat (-33%; p<0.01) and lean (-11%; p<0.01) mass, while food intake and energy expenditure were unaffected. As compared to control mice, BMT-treated mice had a reduced mature adipocyte volume (approx. -45%; p<0.05) and reduced numbers of preadipocytes (-38%; p<0.05) and macrophages (-62%; p<0.05) in subcutaneous, gonadal and visceral white adipose tissue. In BMT-treated mice, pancreas weight (-46%; p<0.01) was disproportionally decreased. This was associated with reduced plasma insulin (-68%; p<0.05) and C-peptide (-37%; p<0.01) levels and a delayed glucose clearance in BMT-treated mice on HFD as compared to control mice. In conclusion, the reduction in HFD-induced obesity after BMT in mice is at least partly due to alterations in the adipose tissue cell pool composition as well as to a decreased pancreatic secretion of the anabolic hormone insulin. These effects should be considered when interpreting results of experimental BMT in metabolic studies.

## Introduction

Overweight and obesity are presently affecting close to 50% of the adult population in many Western countries and are leading to an epidemic of associated metabolic comorbidities such as type 2 diabetes and cardiovascular diseases [1]. Obesity is associated with adipose tissue inflammation, characterized by abnormal production of pro-inflammatory cytokines and chemokines by adipocytes and infiltrating immune cells, which ultimately leads to chronic systemic inflammation [2]. Most of the immune cells are derived from bone marrow [3]. A widely used tool to study the role of immune cells in many immune-associated disorders is experimental bone marrow transplantation (BMT) in mouse models. With this technique, host hematopoietic cells are depleted by lethal total body irradiation (TBI) and replaced by donor bone marrow cells harbouring genetic alterations in a relevant inflammatory pathway [4].

Experimental BMT in mice is relatively easy, effective and cost-efficient. However, a drawback is that BMT, and in particular the lethal TBI that is part of the procedure, may induce metabolic disturbances *per se*. Poglio *et al*. [5] showed that mice have a lower body weight seven days post BMT and that irradiation dose dependently and acutely reduced white adipose tissue (WAT) fat pad weight by decreasing both adipocyte volume and number. Another study showed that BMT reduced adiposity in genetically obese *ob/ob* mice. These mice stopped gaining weight two months after TBI, while the control mice gained weight continuously [6].

We set out to investigate the mechanism underlying the metabolic phenotype of mice fed a low-fat normal diet (hereafter called LFD) or high-fat diet (HFD) after BMT, and focused on body composition as well as glucose and insulin metabolism. We show that BMT treatment indeed significantly decreased diet-induced obesity which involves altered characteristics of white adipocytes and a decreased insulin secretion in response to a HFD.

## Material & methods

### Animals

Male C57BL/6 mice were purchased from Charles River (Maastricht, The Netherlands) and housed under standard conditions with free access to water and food. After a period of two weeks acclimatisation, half of the mice underwent syngeneic BMT. In short, mice received 8 Gy X-ray radiation using an Orthovolt and the day thereafter an intravenous injection in the tail with donor bone marrow cells. The donor mice were male C57BL/6 mice from similar age. All mice, both BMT-treated and non BMT-treated control mice, received antibiotics-water (Amfotericine B, Ciprofloxaci, Polymixin B) from 3 days before until 4 weeks after BMT. After 8 weeks recovery on chow diet, mice were fed low-fat normal diet (LFD; 10% energy from lard D12450B) or a HFD (60% energy derived from lard fat D12492, Research Diet Services, Wijk bij Duurstede, The Netherlands) for 16 weeks to induce obesity (n = 7–12 per group). The experiment started with n = 12 per group. During the study one mouse died from the BMT-treated HFD-group within the recovery period and 4 mice were euthanized during the HFD-feeding due to deteriorated general health condition. This resulted at the end in n = 7 mice for the BMT-treated HFD-group and n = 12 mice for the other 3 groups. Body weight was measured weekly during the entire experiment. Lean and fat mass were monitored by MRI-based body composition analysis (Echo MRI, Echo Medical Systems, USA). Twenty-four weeks after BMT, mice were anesthetized by a subcutaneous injection of a mixture of Neurotranq, Midazolam and fentanyl. Mice were bled via the eye and the following organs were dissected: heart, liver, pancreas, thymus, spleen, skeletal muscle quadriceps, and white adipose tissue pads from the gonadal (gWAT, unilateral), subcutaneous (sWAT, unilateral) and visceral (vWAT) region. All experiments were approved by the animal ethics committee of the Leiden University Medical Center (protocol no. 121031).

### Intravenous glucose tolerance test

At 12 weeks LFD or HFD feeding, an intravenous glucose tolerance test (IVGTT) was performed. Prior to the IVGTT, mice were fasted for 6 hours. Blood samples were collected from the tail vein immediately before (t = 0 min) and 2, 5, 15, 30, 90 and 120 minutes after intravenous injection with glucose (2 mg/g body weight). Plasma glucose concentrations were quantified using the Glucose Start Reagent Method according to manufacturer’s instructions (Instruchemie, Delftzijl, The Netherlands). Plasma insulin and C-peptide levels were measured at 12 weeks LFD or HFD using the Ultra Sensitive Mouse Insulin ELISA Kit and Mouse C-Peptide ELISA Kit, respectively, according to manufacturer’s instructions (Crystal Chem, Downers Grove, USA).

### Adipose tissue characterization

Adipose tissue from the gWAT (unilateral), sWAT (unilateral) and vWAT region were removed from the mice after 16 weeks of LFD or HFD and kept in PBS. The tissues were minced, digested with 0.5 g/l collagenase (Type 1) in DMEM/F12 with 20 g/l of dialyzed bovine serum albumin for 1 h at 37°C (BSA, fraction V; Sigma, ST Louis, USA), and filtered through a nylon mesh (236 μm pore). Adipocytes were obtained from the surface of the filtrate, and washed two times with PBS. Cell size and volume of mature adipocytes were determined from micrographs (approx. 1,000 cells per WAT sample) using image analysis software that was developed in house in MATLAB (MathWorks, Natick, MA). The adipocyte number per fat pad was calculated from the fat pad mass and adipocyte size.

Stromal vascular cells were isolated from the adipose tissue filtrate and fixed in 0.5% paraformaldehyde as described [7]. The number of stromal vascular cells per fat pad was determined using an automated cell counter (TC10, Biorad, CA, USA). The percentage of preadipocytes within the stromal vascular fraction was measured using flow cytometry. The stromal vascular cells were stained with fluorescently labeled antibodies for CD45, CD31, CD34 and F4/80 (BioLegend, CA, USA). Cells were measured on an LSR II flow cytometer (BD Biosciences, Breda, the Netherlands). Data was analyzed using FlowJo software (FlowJo, Oregon, USA). Preadipocytes were determined by selecting the CD45-CD31-CD34+ cells. Macrophages were determined by selecting the CD45+F4/80+ cells.

### Histological examination of the pancreas

The pancreas was isolated, weighed and fixed in a random orientation in 4% paraformaldehyde and embedded in paraffin. Beta cell area and mass of n = 5 mice per group were quantified as described before [8]. For the identification of beta cells, sections were immunostained with rabbit anti-insulin IgG (1:200 dilution; Santa Cruz Biotechnology, Santa Cruz, CA, USA) for 1 hour followed by HRP- or AP-conjugated secondary antibodies (1:100 dilution) for 1 hour. Sections were developed with 3,3’-diaminobenzidine tetrahydrochloride (DAB) and counterstained with hematoxylin. For determining the beta cell mass, 3–4 insulin-DAB stained sections (200 mm apart) were digitally imaged (Panoramic MIDI, 3DHISTECH, Hungary). The area of the beta cells within the pancreas were determined using an in house developed image analysis program (Stacks 2.1, LUMC, Leiden, The Netherlands), excluding large blood vessels, larger ducts, adipose tissue and lymph nodes. The area of clusters containing beta cells was individually measured and used to determine the average beta cell cluster area. Beta cell mass was determined by the percentage of beta cell area to pancreas area multiplied by the total pancreas weight, as described previously [8].

### Faecal triglycerides and free fatty acids measurement

Faecal samples were weighted and manually pulverized. Faecal powder was dissolved in a mixture of MilliQ water, methanol and hexane. Samples were vortexed, centrifuged at 14,000 g for 1 minute and the hexane supernatant was transferred into a clean tube. Additional hexane was added. After again vortexing and centrifuging at 14,000 g for 1 minute, the hexane fractions were transferred into new clean tubes. The hexane was evaporated using N2. Triton-chloroform was added to the pellets and the samples were incubated 10 minutes at 37°C. Triglycerides were measured using the enzymatic kit 11488872 (Roche Molecular Biochemicals, Indianapolis, IN, USA). Free fatty acids were measured using Wako NEFA-C kit from Wako Diagnostics (Instruchemie, Delfzijl, The Netherlands).”

### Statistics

Data are presented as means ± SD. BMT-treated mice were compared to control mice using unpaired student T-test analysis. Correlation analysis was performed using linear regression analysis. The regression lines of the BMT-treated mice versus control mice were compared to see whether the correlations differed between BMT-treated and control mice. First it was tested whether slopes of the lines differed and then whether intercepts of the lines differed. When the slopes and intercepts were not significantly different, linear regression analyses was performed on pooled data of both groups,. All statistical analyses were performed using GraphPad Prism version 6 (GraphPad software, San Diego, CA, USA).

## Results

### After LFD or HFD, body weight gain, but not energy expenditure and food intake, is decreased in BMT-treated mice

**Fig 1:**
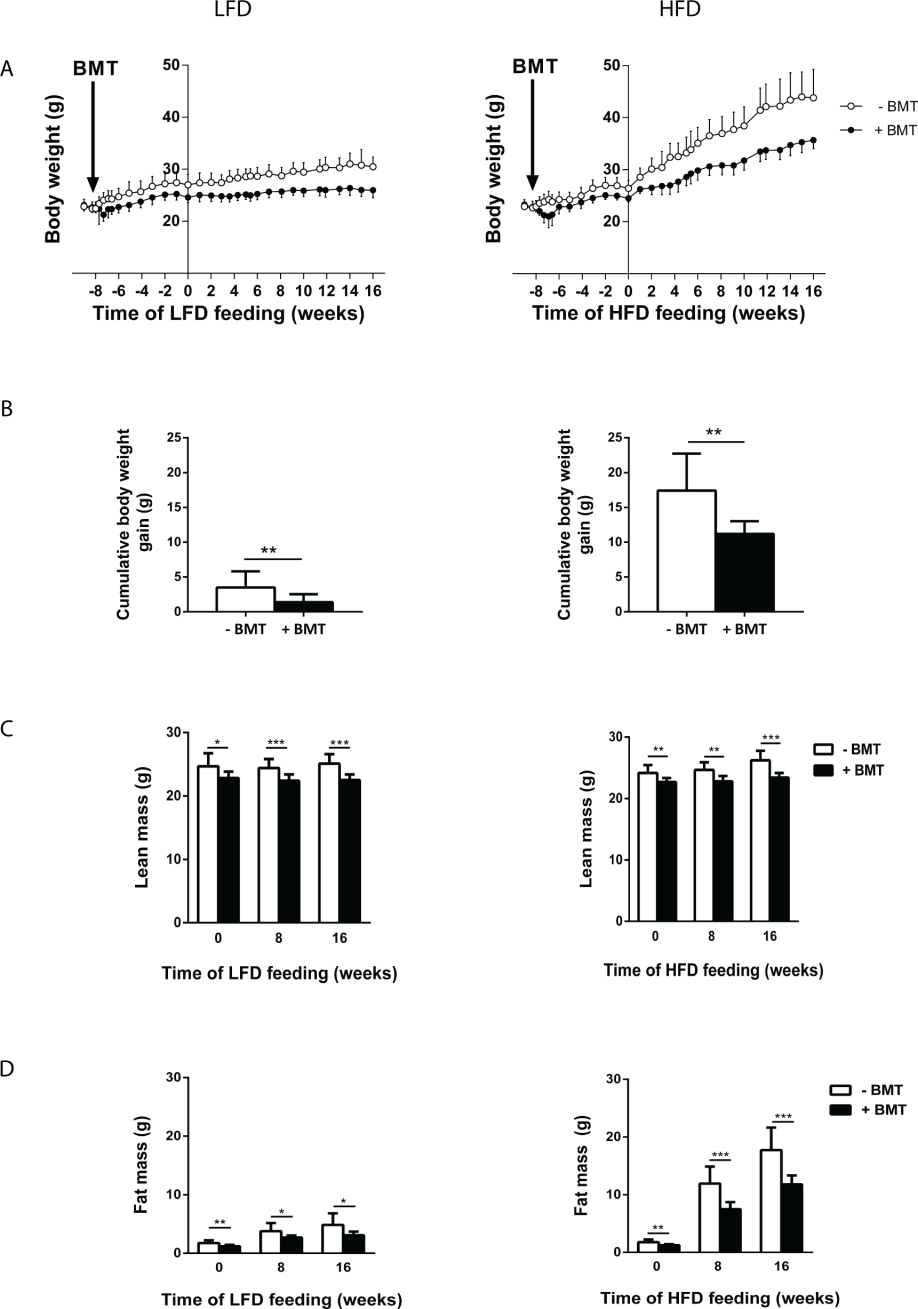
BMT decreased diet-induced obesity. (A) Male C57BL/6 mice underwent BMT at time point 0 weeks. From 0 weeks until week 8, mice were fed a chow diet and from time point 8 weeks, mice were fed either a 10% LFD. (B) Body weight gain of BMT-treated mice, over a period of 16 weeks LFD, was reduced compared to control mice. Values are means ± SD; n = 7–12; * p<0.05,** p<0.01, *** p<0.001.

We first determined the effects of LFD or HFD on body weight gain and body composition as well as on food intake and energy expenditure in mice that had undergone a syngeneic BMT versus controls. The BMT procedure caused a reduction in body weight already within a few days after BMT while mice were fed chow diet (Fig 1A and 1B). After the diet switch to LFD or HFD at t = 0 weeks, the body weight gain diverged between BMT-treated and control mice (Fig 1A and 1B). This was most apparent for HFD-fed BMT mice. Analysis of body composition by Echo-MRI showed decreased lean mass (Fig 1C) and fat mass (Fig 1D) both on LFD and HFD in BMT-treated mice as compared to controls. However, the decreased body weight gain was mainly due to decreased fat mass expansion, especially in the HFD-fed BMT-treated mice. Individual indirect calorimetry measurements using metabolic cages revealed that the decrease in body weight gain could neither be explained by decreased food intake nor by increased energy expenditure in LFD- or HFD-fed BMT-treated mice. There was no difference in fat oxidation or carbohydrate oxidation between BMT-treated and control mice (S1 Fig).

### LFD- and HFD-induced increases in white adipose tissue weight, adipocyte size and macrophage infiltration are diminished by BMT treatment

**Fig 2:**
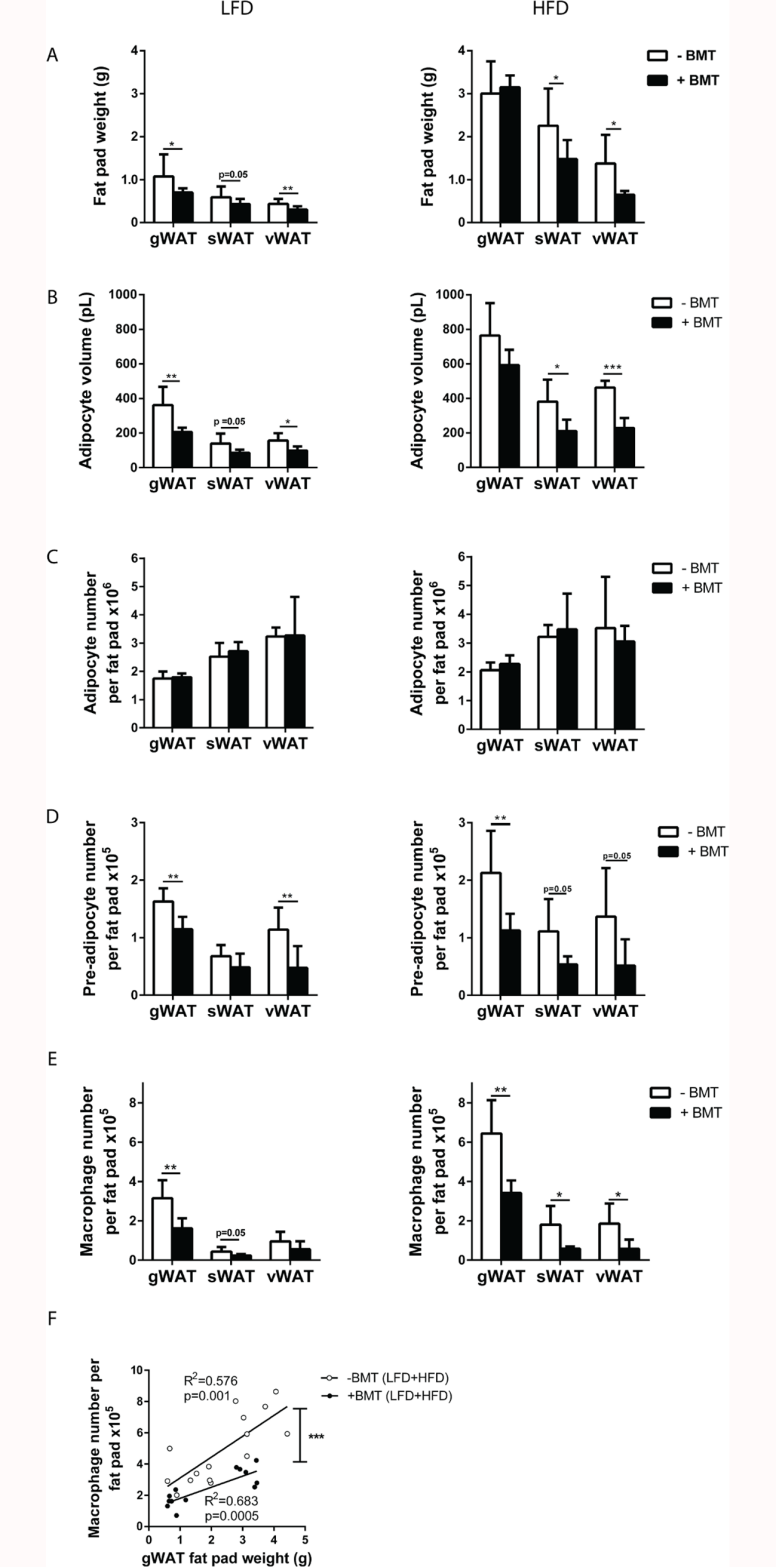
BMT has long term effects on white adipose tissue. (A) Weight of sWAT and vWAT pads was decreased in BMT-treated mice as compared to control mice. (B) The volume of mature adipocytes was lower in BMT-treated mice than control mice. (C) The number of mature adipocytes was not affected in the three WAT pads, (D) but the number of preadipocytes in all three WAT pads was lower in BMT-treated mice than control mice. (E) Macrophage number per fad pad was lower in the three fat pads of BMT-treated mice vs. control mice. (F) Linear regression analysis of macrophage number per fat pad and gWAT pad weight showed decreased macrophage numbers in gWAT in BMT-treated mice compared to control mice. Values are means ± SD; n = 7–12; * p<0.05, ** p<0.01, *** p<0.001.

To investigate whether BMT affects adipose tissue after LFD or HFD, sWAT, vWAT and gWAT of BMT-treated and control mice were characterised. On LFD, the weights of all individual fat pads were reduced at the end of the study in BMT-treated mice as compared to control mice (Fig 2A). On HFD, the weight of sWAT and vWAT, but not gWAT, was reduced in the BMT-treated group as compared to control mice (Fig 2A). The adipocyte volumes in the various fat pads decreased in parallel with decreased fat pad weights (Fig 2B). The number of mature adipocytes per fat pad was not affected by BMT in the WAT pads neither on LFD nor on HFD (Fig 2C). However, FACS analyses revealed that in BMT-treated mice the absolute number of preadipocytes present in adipose tissue was lower in all fat pads on HFD and in the gWAT and vWAT on LFD, than in the controls (Fig 2D). In addition, the absolute numbers of macrophages per fat pad in almost all fat pads in mice on LFD and HFD was lower in BMT-treated mice than in controls (Fig 2E). A clear correlation was observed between gWAT fat pad weight and macrophage number per fat pad (Fig 2F). Interestingly, at similar fat pad weight, the number of macrophages was lower in BMT-treated mice than in control mice, as demonstrated by the downward shifted correlation line (comparison regression lines: slopes F_slopes_ = 2.91, p = NS, F_intercepts_ = 18.49; p<0.01). Collectively, these findings show that LFD- and HFD-induced WAT expansion is significantly impaired after BMT treatment.

### The weight of non-adipose tissue organs in LFD- and HFD-fed mice is decreased after BMT treatment

**Fig 3:**
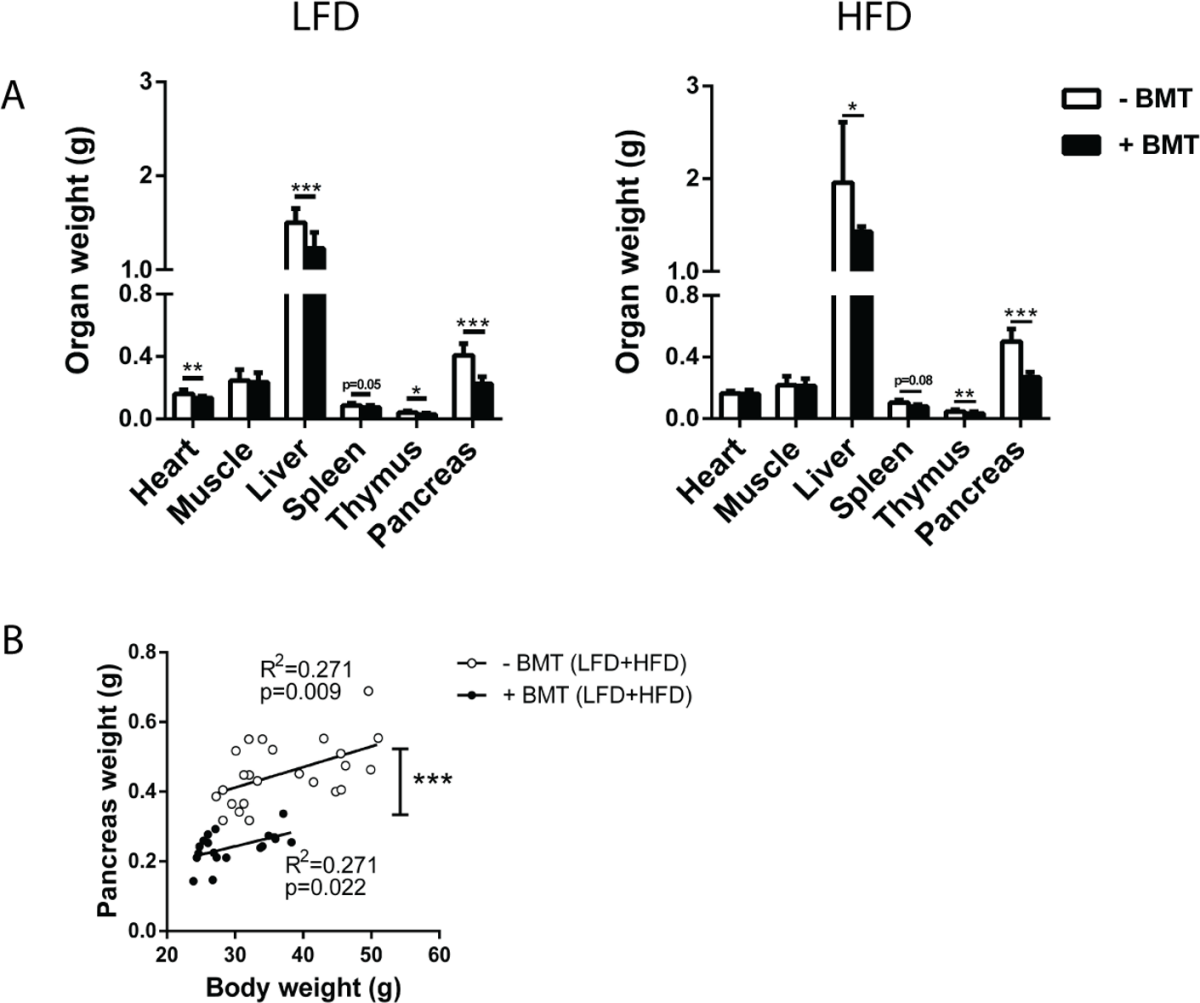
BMT decreased weight of different organs. (A) BMT decreased the weight of liver, thymus, spleen and the pancreas both on LFD and HFD. (B) Linear regression analysis of pancreas weight and body weight showed a lower pancreas weight in the BMT-treated mice, independently of body weight. Values are means ± SD; n = 7–12; * p<0.05, ** p<0.01, *** p<0.001.

**Fig 4:**
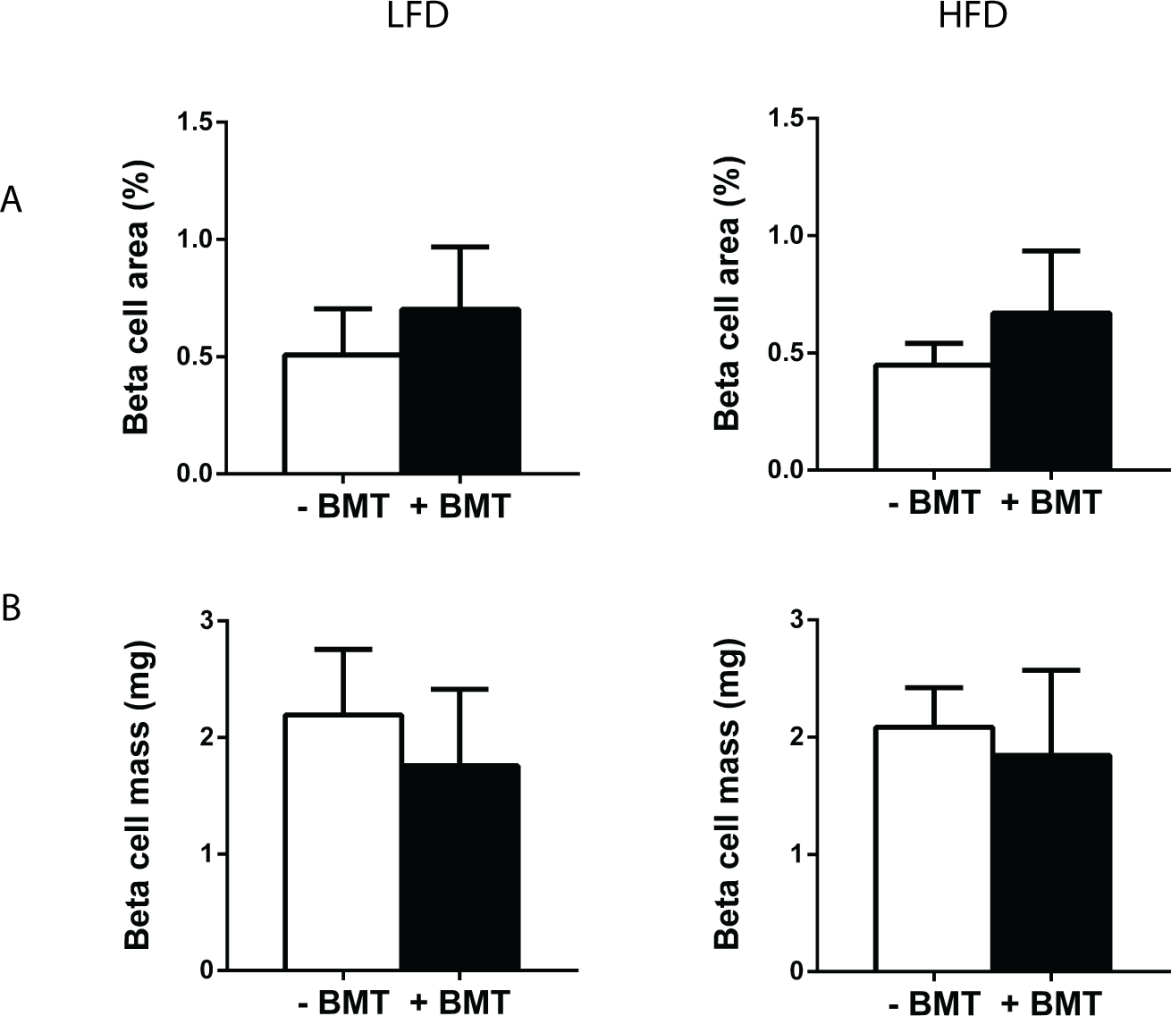
BMT did not affect pancreatic beta cell area and beta cell mass. (A) Beta cell area and (B) beta cell mass were not different in the BMT-treated mice. Values are means ± SD; n = 5.

Further characterisation of organs revealed that in LFD- and HFD-fed mice, weight of liver, thymus and spleen was lower in BMT-treated mice than in controls (Fig 3A). Heart weight was lower in LFD-fed but not in HFD-fed BMT-treated mice. BMT treatment did not affect skeletal muscle quadriceps weight. Remarkably, both in LFD- and HFD-fed mice, pancreas weight was reduced by approx. 45% in BMT-treated mice (p<0.01) (Fig 3A). Liver, thymus, spleen and pancreas weight correlated with body weight (data not shown). However, BMT-treated mice had lower pancreas weights than control mice at similar body weights, as demonstrated by the significantly downward shifted correlation line (comparison regression lines: F_slopes_ = 0.109, p = NS, F_intercepts_ = 56.81; p<0.01; Fig 3B). This difference in organ weight relative to body weight between the mouse groups was not seen for liver, thymus and spleen. The pancreas was further characterized by histological examination. Surprisingly, total beta cell area or beta cell mass per total pancreas was not affected by BMT treatment (Fig 4).

### Insulin production and glucose tolerance are lower in HFD-fed mice after BMT treatment

**Fig 5:**
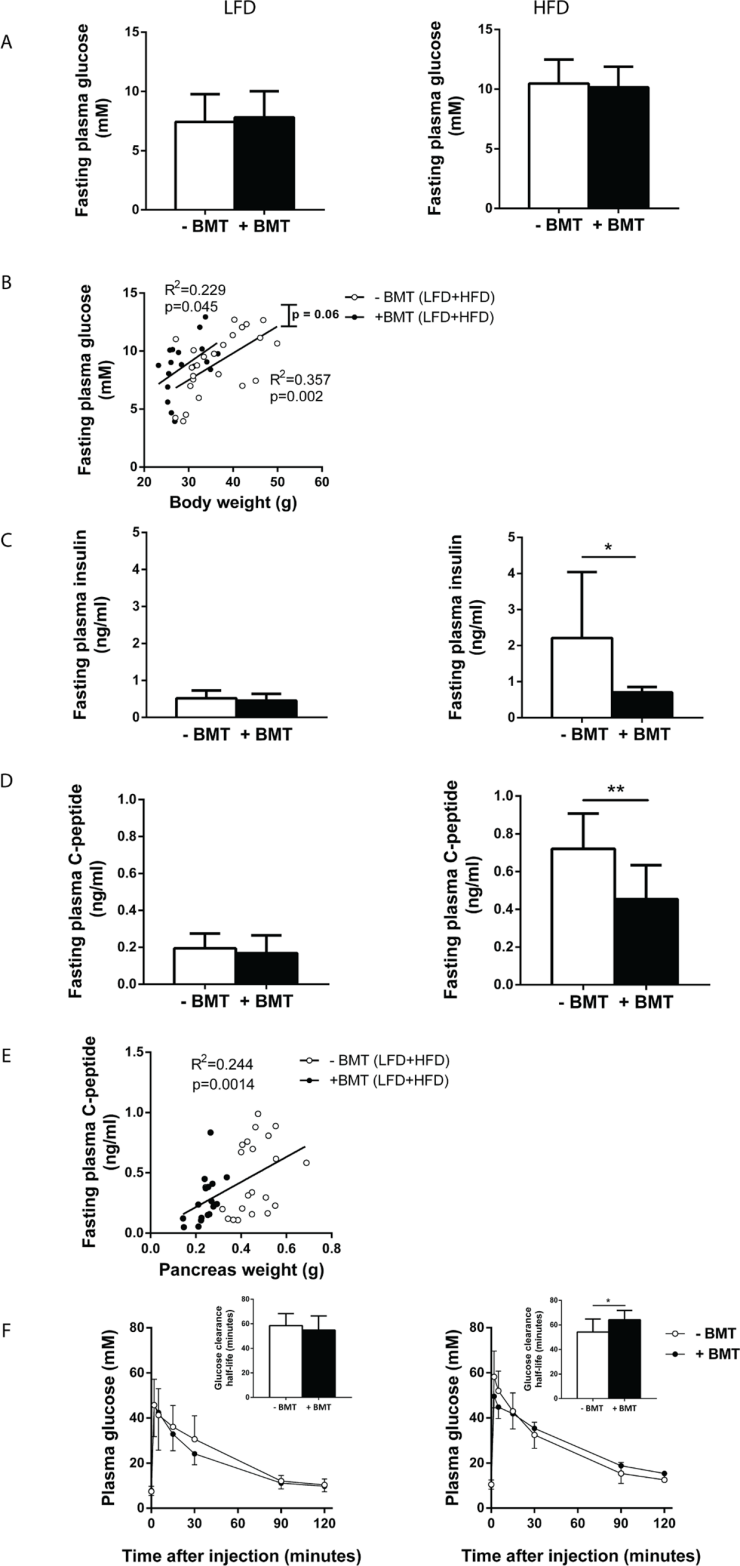
BMT mice had lower plasma insulin levels and glucose tolerance on a HFD. (A) Fasting plasma glucose of BMT-treated and control mice did not differ on LFD or HFD. (B) BMT-treated mice tended to have higher fasting plasma glucose concentrations for similar body weights. (C) BMT-treated mice had lowered fasting plasma insulin levels only on HFD. (D) BMT-treated mice had lowered plasma C-peptide concentrations on HFD. (E) There is a linear relation between pancreas weight and fasting plasma C-peptide concentrations. (F) IVGTT revealed that BMT-treated mice have a higher plasma glucose clearance half-life, only on HFD. Values are means ± SD; n = 7–12; * p<0.05, ** p<0.01.

As pancreas weight relative to body weight was disproportionally reduced in both LFD- and HFD-fed mice after BMT treatment, we further assessed the effect of BMT treatment on glucose and insulin metabolism. Neither on LFD nor on HFD fasting plasma glucose concentrations were affected by BMT treatment (Fig 5A). To investigate whether BMT itself also affected fasting plasma glucose levels independently of body weight, linear regression analysis was performed on fasting plasma glucose levels versus body weight for BMT-treated and control mice separately. BMT-treated mice tended to have a higher fasting plasma glucose concentration with similar body weights (Fig 5B: comparison regression lines: F_slopes_ = 0.065, p = NS, F_intercepts_ = 3.72; p = 0.06). Interestingly, fasting plasma insulin concentration was only lower in BMT-treated mice than in controls when they were fed a HFD (-68%; p<0.05; Fig 5C). These decreased insulin concentrations in the metabolically-challenged BMT-treated mice may be the result of a lower insulin production and, therefore, fasting plasma C-peptide levels were measured. Indeed, BMT reduced fasting plasma C-peptide levels compared to controls only in HFD-fed mice (-37%; p<0.01; Fig 5D). The pancreas weight positively correlated with C-peptide levels (R^2^ = 0.244; p<0.01; Fig 5E), indicating that the reduced pancreas volume in BMT mice was linked to lower insulin secretion. In line with reduced insulin secretion, HFD-fed BMT-treated mice were more glucose intolerant during an IVGTT than control mice, as reflected by an increased glucose clearance half-life (Fig 5F).

## Discussion

The current study shows that BMT induces long-term metabolic abnormalities in mice, with decreased body weight gain upon LFD and, especially, upon HFD feeding. This reduced body weight gain was mainly due to reduced fat mass gain, and to a lesser extent due to reduced lean mass gain. BMT-treated mice displayed decreased adipocyte hypertrophy both on LFD and HFD, whereas adipocyte hyperplasia was not affected 24 weeks after BMT. In addition to reduced adipose tissue, BMT led to a decreased size of other (metabolic) organs such as liver, thymus, spleen and pancreas, which, except for the pancreas, was in line with the reduction in body weight. The pancreas weight disproportionally decreased after BMT and likely explains the decreased plasma insulin levels and insulin secretion on a HFD after BMT.

We observed smaller adipocytes in BMT-treated mice, which could not be explained by a reduced food intake or increased energy expenditure as determined by indirect calorimetry. The smaller adipocytes might be explained by alterations in preadipocyte function. Mature adipocytes are terminally differentiated cells which originate from preadipocytes present in adipose tissue [9–11]. Recently, Nylander et al. [12] showed that a single sub-lethal irradiation dose of 6 Gy attenuated the adipogenic potential of preadipocytes via alterations in intracellular and epigenetic pathways. A reduced preadipocyte differentiation capacity may explain the reduced mature adipocyte volumes in our BMT-treated mice.

Our study revealed that also the number of preadipocytes per fat pad was reduced in the BMT-treated mice, suggesting that the preadipocyte pool used for recruitment of mature adipocytes was decreased by BMT. As both LFD and HFD stimulate energy storage and therefore likely induce preadipocyte recruitment, a decreased preadipocyte pool may also have contributed to the decreased adipose tissue growth seen in BMT-treated mice. The mechanism underlying the lower preadipocyte numbers in BMT-treated mice remains to be determined. It is possible that the TBI procedure has depleted preadipocytes or the mesenchymal stem cells in adipose tissue that are believed to be the precursor cells of the preadipocytes.

Notably, several studies have suggested that progenitor cells derived from bone marrow can contribute to the development of new adipocytes both in mice [13,14] and humans [15]. This population of bone marrow cells may have been affected by the BMT procedure and thus also contribute to the reduced adipogenesis. Infiltration of bone marrow-derived macrophages into WAT tissue is strongly associated with fat pad expansion [16]. Interestingly, the macrophage content in WAT fat pads after BMT was disproportionally reduced. Thus, BMT not only affects the response of adipocyte precursor cells to LFD and HFD, but also the immune cell composition in the adipose tissue. Since immune cells play a major role in adipose tissue biology [16,17], our data imply that BMT may also affect WAT function via modulation of the immune status in this tissue.

The weight of organs is in general strongly associated with body weight. In accordance, obesity induces changes in body composition and increases in organ weights [16]. BMT more severely reduced pancreas weight than expected based on the reduction in body weight both in mice on LFD as well as HFD. This was accompanied by a decreased insulin secretion and decreased plasma insulin levels in BMT mice as compared to control mice on HFD. The fact that we only observed this difference in insulin levels on a HFD is explained by a HFD-induced increase in plasma insulin in control mice which was not seen control mice on LFD. BMT-treated mice were slightly more glucose intolerant than control mice on a HFD, despite a lower body weight. Pancreatic beta cell mass, however, was not affected by BMT treatment. Our data together suggest that BMT disproportionally affects the weight of the pancreas, resulting in reduced insulin responses to a HFD, and modest negative effects on glucose metabolism.

It was previously suggested that BMT leads to β-cell regeneration after acute streptozotocin (STZ)-induced injury in mice by triggering recruitment of immature bone marrow-derived cells to the injured pancreas. This recruitment, subsequently, stimulates stem/progenitor cells located in the recipient pancreas, resulting in islet regeneration. It was therefore suggested that BMT can be used as a novel approach to treat T2D [18]. Our results point to the opposite direction and suggest that BMT may lead to metabolic abnormalities, including reduced insulin secretion and glucose intolerance, which is specifically manifest on HFD. Interestingly, reduced pancreas volume was also observed in survivors of childhood acute lymphoblastic leukaemia who were treated with BMT [19]. In line with our study, several clinical studies suggests that BMT survivors have an increased risk of developing insulin resistance and T2D development [16,10,20].

In addition to producing the anabolic hormone insulin, the pancreas plays a major role in energy metabolism by secreting digestive enzymes such as pancreatic lipases into the gut where they serve to digest dietary lipids. As BMT reduced the pancreas volume, it is possible that the exocrine function of the pancreas was affected. Reduced absorption of lipids may thus in part explain the reduced body fat gain in BMT-treated mice. To elucidate whether there were differences in pancreatic lipid digestion, we measured faecal triglyceride and free fatty acids content. For faecal triglycerides we did not find differences between the BMT-treated and control mice, whereas for faecal free fatty acids there was a slight decrease in BMT-treated mice on HFD (S2 Fig). These results indicate that pancreatic lipase production was not affected significantly by BMT. Whether BMT affects the function of other digestive pancreatic enzymes (such as pancreatic proteases) remains to be elucidated.

Taken together, we conclude that the BMT procedure has multilevel effects on the organism and affects metabolically important organs. These multi-level effects are associated with metabolic abnormalities that involve both WAT and pancreas dysfunction in response to a HFD. While BMT is a relatively easy and effective strategy for assessing the role of specific immune-related genes in pathophysiological studies in mice, the metabolic effects of BMT cannot simply be neglected and should be seriously considered in study design, goal and interpretation of the data. Our study may also have clinical implications, as BMT is conducted in humans to treat diseases such as leukaemia. Weight loss and insulin resistance should be closely monitored as potential side effects of this therapy.

## Supporting information

S1 Fig BMT did not affect food intake, energy expenditure, fat and carbohydrate oxidation. Indirect calorimetry data using metabolic cages (Phenomaster, TSE Systems, Bad Homburg, Germany) showed (A) Food, (B) energy expenditure, (C) fat oxidation and (D) carbohydrate oxidation of BMT-treated mice did not differed compared to control mice.
(TIF)

S2 Fig BMT did not affect faecal triglycerides content. (A) Faecal triglycerides concentration did not differed between BMT-treated and control mice. (B) There was slightly less free fatty acids in feces of HFD fed BMT-treated mice compared to the control group.
(TIF)
