# Supplementary figures and images for: BMT decreases HFD-induced weight gain associated with decreased preadipocyte number and insulin secretion
Source: PLoS One. 2017 Apr 26;12(4):e0175524. doi: 10.1371/journal.pone.0175524 (PMC5406023; doi:10.1371/journal.pone.0175524)

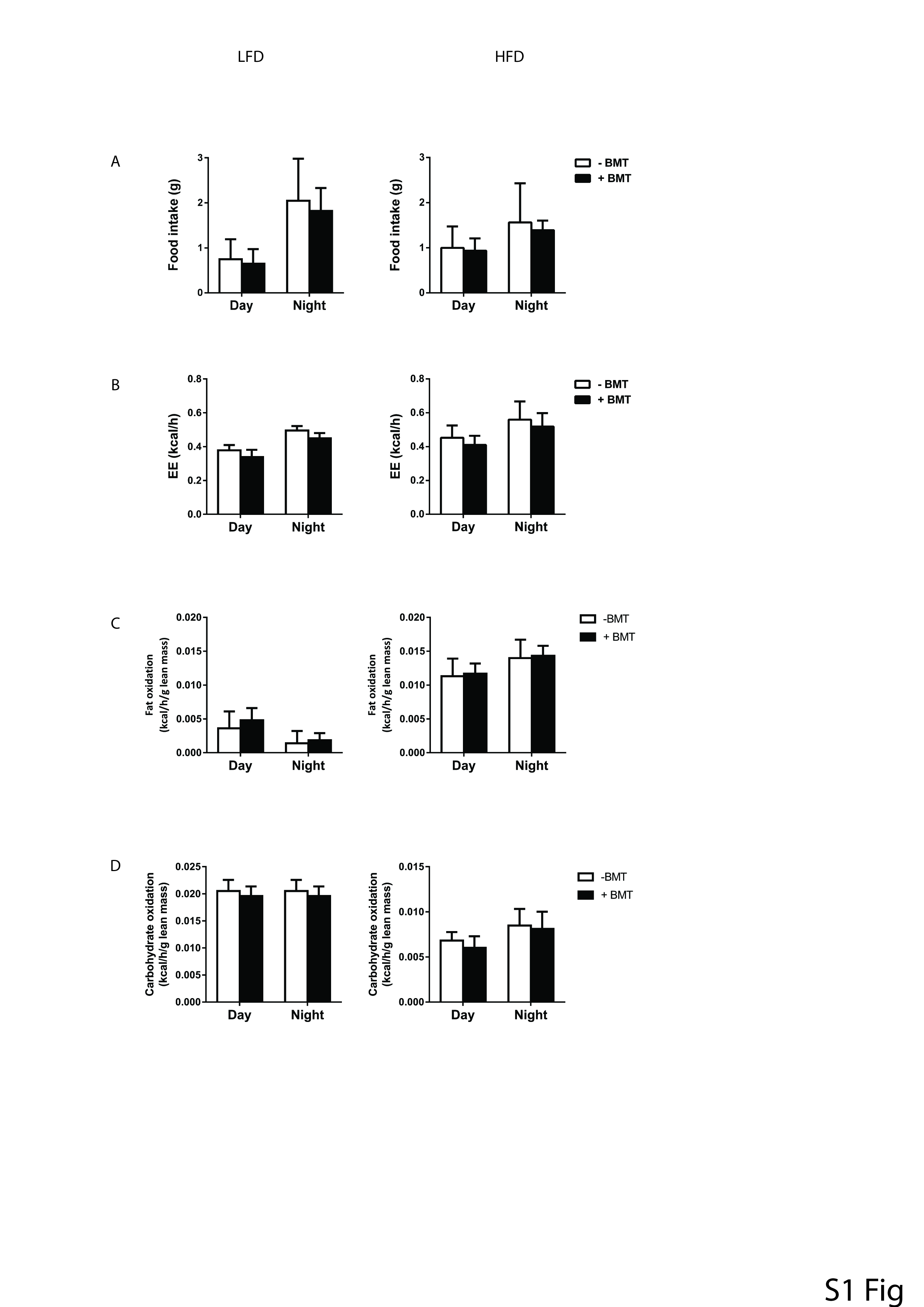

Supplement: S1 Fig — Indirect calorimetry data using metabolic cages (Phenomaster, TSE Systems, Bad Homburg, Germany) showed (A) Food, (B) energy expenditure, (C) fat oxidation and (D) carbohydrate oxidation of BMT-treated mice did not differed compared to control mice. (TIF) [file pone.0175524.s001.tif]

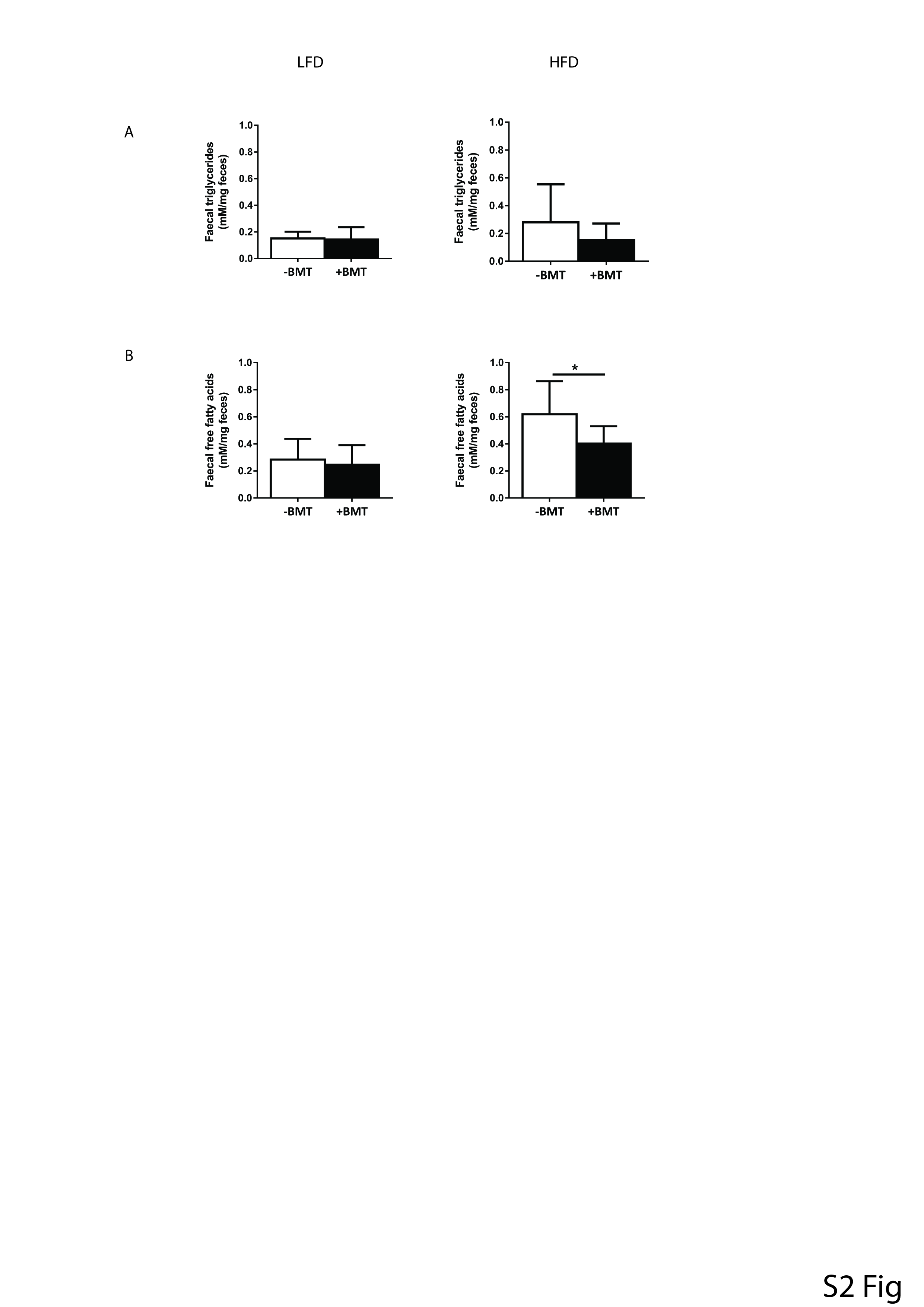

Supplement: S2 Fig — (A) Faecal triglycerides concentration did not differed between BMT-treated and control mice. (B) There was slightly less free fatty acids in feces of HFD fed BMT-treated mice compared to the control group. (TIF) [file pone.0175524.s002.tif]
